# Transcranial Focused Ultrasound Stimulation for Alzheimer’s Disease—A Scoping Review

**DOI:** 10.3390/brainsci16060570

**Published:** 2026-05-28

**Authors:** Jon Crompton, Robyn Cuthell, Tom G. J. Steward, William W. Watts, Alanoud Alqahtani, Daniel J. Whitcomb

**Affiliations:** School of Psychology and Neuroscience, University of Bristol, Bristol BS8 1TD, UK; xf20228@bristol.ac.uk (J.C.); robyn.cuthell@bristol.ac.uk (R.C.); ts7764@bristol.ac.uk (T.G.J.S.); william.w.watts@bristol.ac.uk (W.W.W.); qx19627@bristol.ac.uk (A.A.)

**Keywords:** Alzheimer’s disease, tFUS, LIPUS, focused ultrasound, neuromodulation, non-invasive brain stimulation, synaptic plasticity, neurodegeneration

## Abstract

**Highlights:**

**What are the main findings?**

- Clinical and preclinical Alzheimer’s disease studies confirm that transcranial focused ultrasound stimulation (tFUS) is safe and feasible, inducing measurable improvements in memory and cerebral metabolism without the need to disrupt the blood–brain barrier.
- The mechanisms of tFUS actions in Alzheimer’s disease continue to be characterised, but current evidence suggests reductions in amyloid-β and tau burden via mechanical disruption and microglial modulation, with concurrent restoration of dysregulated gamma oscillations.

**What are the implications of the main findings?**

- tFUS represents a promising multi-modal intervention that uniquely addresses both the biochemical (protein aggregation) and electrophysiological (network connectivity) deficits of Alzheimer’s disease.
- The observation of cognitive improvement independent of plaque clearance challenges the amyloid cascade hypothesis and suggests a novel therapeutic pathway driven by neurotrophic upregulation and synaptic plasticity.

**Abstract:**

**Background/Objectives**: Alzheimer’s disease (AD) remains a significant global health challenge, characterised by a persistent resistance to traditional pharmacological interventions. While non-invasive brain stimulation (NIBS) techniques like transcranial magnetic stimulation (TMS) and transcranial direct current stimulation (tDCS) show therapeutic promise, their limited depth of penetration restricts their efficacy in targeting deep-brain AD pathology. Transcranial focused ultrasound stimulation (tFUS) has emerged as a novel, non-invasive neuromodulatory tool capable of precise deep-brain targeting. This scoping review aims to systematically map the current evidence base regarding the neuromodulatory application of tFUS in AD. **Methods**: Following PRISMA-ScR guidelines, a scoping search was conducted across four major databases (Ovid MEDLINE, Embase, Web of Science, and CENTRAL). Studies were included if they investigated focused ultrasound stimulation (FUS) as a neuromodulatory intervention for AD, excluding applications involving blood–brain-barrier disruption via microbubbles. Two independent reviewers performed screening and data extraction, with inter-rater reliability assessed via Cohen’s kappa. **Results**: Our analysis indicates that tFUS represents a safe and potent multi-modal intervention for AD that addresses both pathological protein aggregation and electrophysiological network failure. Its ability to modulate neuroplasticity and metabolic recovery suggests a promising therapeutic trajectory. **Conclusions**: Future research should prioritise the standardisation of acoustic protocols and the pursuit of longitudinal clinical cohorts to establish the long-term efficacy of this non-invasive technology.

## 1. Introduction

Alzheimer’s disease (AD) is the leading form of dementia, accounting for around 60% of the clinical population [1]. Characteristic symptoms of AD are progressive memory loss and development of difficulties with language, perception, and problem solving. Continuing biochemical and neuroanatomical study of AD has led to significant advances in our understanding of the underlying pathology of the disease, and the role of amyloid beta (Aβ) aggregation and tau hyperphosphorylation is well established [2]. Despite this, relatively few therapeutic advances have been made, and the disease remains resistant to pharmacological treatment [3,4].

In the search for efficacious interventions, attention has recently turned toward non-pharmacological approaches. Leading amongst these is non-invasive brain stimulation, which describes a range of techniques designed to modulate neural function non-invasively. Several distinct techniques are used within this context, including transcranial magnetic stimulation (TMS) and transcranial direct current stimulation (tDCS), which leverage magnetic and electrical field energy, respectively [5,6]. The growing evidence supporting the therapeutic potential of these approaches is highly encouraging. For instance, a number of studies indicate that TMS has a positive impact on cognitive performance in both AD patients and its precursor, Mild Cognitive Impairment (MCI) [7]. However, the spatial focality and limited penetration depth of these approaches make these technologies unsuitable for stimulation of deep brain structures, where AD pathology typically develops and is at its most severe [8,9].

**Figure 1:**
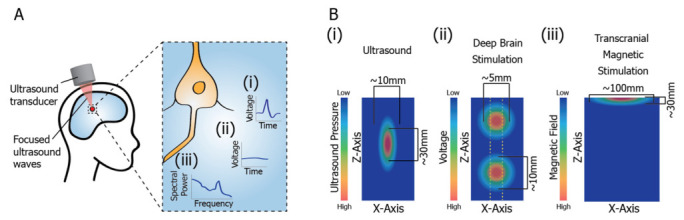
Schematic diagram of neuromodulation by ultrasound. (**A**). An ultrasound transducer is placed on the skull; ultrasonic waves are delivered. Effects (determined by the specifics of the stimulation protocol utilised) include (**i**) increased neuronal firing (**ii**) suppressed neuronal firing (**iii**) modified neural oscillations. (**B**). Representation of typical stimulus focal spots induced by (**i**) ultrasound, (**ii**) deep brain stimulation (DBS) (dashed yellow line represents the DBS electrode shaft), and (**iii**) transcranial magnetic stimulation.

Transcranial focused ultrasound stimulation (tFUS) is an emerging non-invasive and non-ionising technique that can reversibly modulate neural function with precise spatial resolution and provides deep brain penetration [10]. tFUS, therefore, appears to address the limitations associated with the more established brain stimulation techniques (Figure 1) and has been explored as a translational tool for use in several brain-related conditions [11,12,13,14,15]. Whether and how tFUS might be applied in AD, however, remains to be fully determined.

There is good reason to believe that tFUS has the potential to modulate key AD-related brain regions. The hippocampus is a major area of the brain that sees particularly profound dysregulation in AD [8,9]. Encouragingly, several studies now evidence that the hippocampus is sensitive to ultrasound stimulation [16,17,18] Indeed, we have recently evidenced that ultrasound causes sustained enhancement of intrinsic excitability in cortico-hippocampal neurons [19,20]. Furthermore, we have shown that when delivered in vivo, tFUS can be used to condition hippocampal circuits, resulting in metaplastic changes and enhanced magnitudes of subsequently induced long-term potentiation (LTP) form in the CA1 subfield [21].

The precise mechanisms underlying hippocampal neuromodulation by ultrasound, and how best to exploit them for therapeutic benefit, remain to be fully explored. Neuromodulation by focused ultrasound stimulation (FUS) relies on mechanical energy to alter neuronal activity, where mechanosensitive ion channel activation, such as Piezo channels [22], Transient Receptor Potential (TRP) channels [23], voltage-gated Na^+^ channel (Na(v)1.5) [24], two-pore-domain K^+^ channels [24], and Ca^2+^ channels [25], facilitates an increase of inward current across the membrane leading to depolarisation and the generation of action potentials. Several studies have reported that FUS reversibly modulates synchronised neural activity as measured by electroencephalography and magnetoencephalography [26,27,28,29,30]. Importantly, activation of Ca^2+^ channels by FUS promotes the influx of Ca^2+^ intracellularly, initiating signalling cascades that leads to the expression of genes for neurotropic factors [31]. Indeed, the hippocampal kinome appears highly sensitive to FUS, where various kinases are activated, including Ca^2+^/calmodulin-dependent protein kinase II (CaMKII) and AKT Serine/Threonine Kinase (AKT), as a result of stimulation [21]. FUS also interacts with the broader neurovascular unit, including glial cells. FUS can activate astrocytes through the opening of TRPA1 Ca^2+^ channels leading to the release of gliotransmitters such as glutamate through Best1, which modulates synaptic activity [32], and emerging evidence is pointing towards the efficacy of FUS in preventing demyelination and enhancing remyelination in models of demyelinating disease [33,34]. Accordingly, there is significant scope for effects such as these to be leveraged to address AD pathology.

To determine potential future use of FUS neuromodulation in AD, we performed a scoping review of the current literature surrounding this application. The aims were to explore the current approaches and methods in this research in a formal manner [35]. Our intent is to review current approaches and identify potential gaps in existing knowledge that might guide future preclinical and translational research.

## 2. Materials and Methods

This scoping review adheres to the guidelines established by the Preferred Reporting Items for Systematic Reviews and Meta-Analysis Extension for Scoping Reviews (PRISMA-ScR) [35]. In contrast to systematic reviews, the registration of a protocol is not a mandatory requirement for scoping reviews [36]. Given the shortage of comprehensive reviews and the significant heterogeneity in reporting within this field, a scoping review approach was deemed most appropriate to systematically map the emerging evidence regarding the application of tFUS in AD.

### 2.1. Search Strategy

A specific search string was constructed to capture original research examining reversible FUS as a neuromodulatory intervention for AD. Applications involving clinically available high-intensity, ablative FUS were excluded from the scope of this review. The search strategy incorporated synonyms for two primary concepts: “ultrasound neuromodulation” and “Alzheimer’s Disease”. This strategy was executed across four major electronic databases: Ovid MEDLINE, Embase, Web of Science, and the Cochrane Central Register of Controlled Trials (CENTRAL). Searches were performed on each database on 14 January 2026. To ensure the literature was covered comprehensively, no restrictions were placed on publication date or language (Supplementary Table S1).

### 2.2. Study Selection and Reliability

Studies were considered eligible for inclusion if they were published in peer-reviewed journals in any language and explicitly investigated FUS as a neuromodulatory tool within the context of AD (Supplementary Table S2). Two independent reviewers (JC and RC) screened all titles and abstracts against pre-established eligibility criteria. Following this, potentially relevant studies underwent full-text analysis. To ensure literature saturation, the reference lists of included articles were manually scanned to identify further relevant records. Any discrepancies regarding study inclusion were resolved via consensus or through adjudication by a third senior reviewer (DJW). Inter-rater reliability regarding study inclusion was statistically assessed using Cohen’s kappa [37].

### 2.3. Data Extraction

Systematic data extraction was facilitated using a standardised proforma. The data fields captured included the following: (i) study design, (ii) country of origin, (iii) participant details or subject models, (iv) specifics of the intervention, (v) comparator groups, (vi) data collection methods, outcome measures, (vii) and key findings. Data charting was performed independently by two reviewers (JC and RC) for each eligible article. Consistency was ensured by resolving any disagreements through discussion or consultation with a third reviewer (DJW).

### 2.4. Outcomes

Reflecting the exploratory rather than hypothesis-driven nature of this scoping review, outcome variables were not predefined. This approach permitted the inclusion and analysis of a diverse array of behavioural and physiological responses pertinent to the pathology of AD.

### 2.5. Synthesis of Results

Data were synthesised narratively, accompanied by descriptive analyses where applicable, to elucidate relationships both within and between studies and identify gaps in the current literature. We profiled the characteristics of selected studies including location and model type, followed by a thematic analysis to categorise findings into key biological and clinical domains such as amyloid clearance, oscillatory entrainment, and behavioural outcomes.

## 3. Results

### 3.1. Characteristics of Included Studies

**Figure 2:**
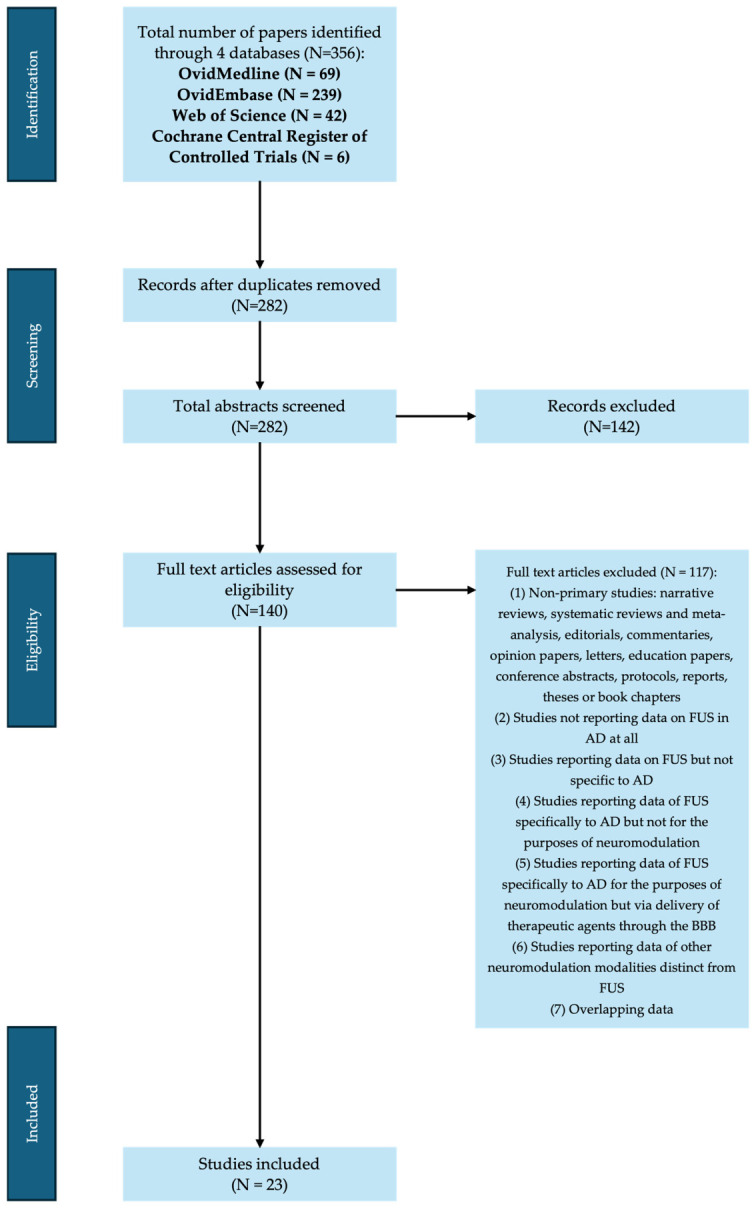
PRISMA flow diagram for studies included and excluded from the scoping review.

Of the 356 unique articles that were identified, 23 were included in the final dataset (Figure 2). Any articles focusing on transcranial pulse stimulation (TPS) were excluded on the basis that TPS is a similar but distinct modality to that of FUS. Articles were excluded if FUS was used in conjunction with microbubbles as a tool to increase the permeability of the blood–brain barrier (BBB). The focus of this review is therefore concerned with the use of transcranial ultrasound stimulation for neuromodulation of the central nervous system within the context of AD as opposed to neuromodulation achieved by the delivery of therapeutic agents.

Inter-rater reliability was assessed using Cohen’s kappa coefficient for 356 citations. The calculated kappa value at the abstract screening stage (Cohen’s κ = 0.96) and the full-text review stage (Cohen’s κ = 1.00) were both high, indicating agreement. Disagreements were resolved by consensus or consultation with a third reviewer (DJW). Among the final 23 articles, 6 were from China, 2 from the United States of America, 5 from South Korea, 3 from Australia, 4 from Taiwan, and 3 from Japan. A total of 16 of the 23 articles were pre-clinical, and 7 were clinical.

### 3.2. Outcome Measures

These studies investigate the therapeutic potential of FUS for treating AD in both human patients and animal models (Table 1). Clinical studies demonstrate that sonication can safely enhance cognitive performance, memory, and functional brain connectivity without necessarily requiring the disruption of the blood–brain barrier. Complementary preclinical research suggests that FUS may reduce Aβ plaques by modulating microglial activity and shifting the brain toward an anti-inflammatory state. Furthermore, advanced proteomic analyses further reveal that these interventions can facilitate favourable molecular changes related to synaptic plasticity and cellular metabolism. Further novel emerging experimental techniques also explore using piezoelectric materials to mechanically break down toxic protein aggregates under ultrasound stimulation. Collectively, these sources present FUS as a promising non-invasive neuroprotective strategy that may slow neurodegeneration through diverse mechanical and biological mechanisms. The acronyms tFUS, FUS, LIPUS, LIFU, and UBDS are used interchangeably throughout this section to conserve author preference but should, unless otherwise stated, be interpreted to have the same meaning.

### 3.3. Findings from In Vitro Preclinical Studies

In vitro preclinical studies focus on the direct mechanical and chemical disruption of Aβ aggregates, the attenuation of Aβ-induced neurotoxicity, and the optimisation of ultrasound parameters for clinical translation. Research using synthetic Aβ_1-42_ aggregates demonstrates that FUS can physically break down otherwise highly stable protein structures. FUS treatment using a 500 kHz single element transducer with a 100 ms pulse duration (PD), 10% duty cycle (DC), 1 Hz pulse-repetition frequency (PRF), 1 s pulse repetition interval (PRI), 1.75, 5, and 12 W/cm^2^ spatial-peak pulse-average intensity (I_SPPA_) for a 30-min pulse train duration (PTD) reduced the β-sheet structure of synthetic Aβ_1–42_ aggregates by up to 55.28% and fibrillar levels by up to 62.27% [40]. Transmission electron microscopy (TEM) confirmed that FUS fragments dense bundles of fibrils into smaller, dispersed particles [40]. Critically, FUS alone without drugs or microbubbles reduced oligomeric Aβ_1-42_ levels by up to 65.02%, indicating it does not merely break fibrils into smaller toxic species but reduces the overall load of neurotoxic oligomers [40].

In a separate study, piezoelectric materials like BiOCl nanosheets were found to be biocompatible with Neuro2a cells, maintaining high viability and proliferative activity over seven days of culture. When Neuro2a cells were exposed to toxic Aβ fibrils pre-treated with sono-activated BiOCl nanosheets, their viability recovered to 85.9%, compared to only 57.1% when exposed to untreated fibrils, suggesting the sono-activated nanosheets provided neuroprotection against the neurotoxicity of Aβ fibrils [46]. Furthermore, BiOCl nanosheets were shown to disassemble Aβ fibrils [46]. These nanosheets generate an internal electric field that separates electron-hole pairs to trigger redox reactions, producing reactive oxidative species (ROS) such as hydroxyl radicals and hydrogen peroxide [46]. These ultrasound-induced ROS oxidise amino acid residues (like histidine and methionine) in Aβ monomers, which weakens the hydrophobic interactions and hydrogen bonds that hold the aggregate structure together [46]. Together, these data point towards the potential of leveraging the mechanical effects of ultrasound to disrupt protein aggregates, a fundamental aspect of AD pathology.

Interestingly, in vitro models using human and mouse cell lines build on this and support the notion that ultrasound treatment renders Aβ aggregates less harmful to neurons. SH-SY5Y human neuroblastoma cells treated with FUS-exposed Aβ_1-42_ aggregates showed improved cell viability in the CCK assay, increasing from 81.56% to 90.48%, suggesting that ultrasound attenuates Aβ-induced cytotoxicity and promotes cell survival [40,46]. This supports a proof-of-principle that sonicated protein aggregates can reduce their biotoxicity.

Beyond the direct effects of ultrasound on Aβ aggregates, in vitro studies have also explored how ultrasound and blood-borne factors influence microglial behaviour. Ultrasound stimulation has been observed to modulate microglial polarisation in vitro, biasing cells away from the pro-inflammatory M1 phenotype toward the neuroprotective, phagocytic M2 phenotype. This shift is associated with an increase in the anti-inflammatory factor IL-10 and a decrease in TNF-α [52]. This attenuation of inflammatory responses and promotion of tissue repair are undoubtedly significant contributors to improving AD pathology and cognitive decline.

Benefitting from these aggregate-disrupting effects of ultrasound will require overcoming a major challenge in the field, however—developing sonication protocols and approaches able to penetrate the thick human skull. Accordingly, optimal stimulation parameters must first be evidenced to penetrate the approximate 5–6 mm thickness of the human skull. Within this context, to translate pre-clinical findings from thin-skulled mice to humans, researchers used human temporal bone and (HUVEC) cultures to identify ideal acoustic conditions. At a carrier frequency of 0.5 MHz, mRNA expression of essential growth factors (VEGF, FGF2, and eNOS) was significantly upregulated at a 20% duty cycle, while a 1% duty cycle was insufficient [47]. Research identified that a tissue amplitude of 0.05–0.5 MPa is effective for upregulating these neurotrophic factors [47]. Conversely, sound pressures consistently exceeding 1.08 MPa or duty cycles above 40% were found to be highly cytotoxic and could reverse therapeutic benefits [47]. This HUVEC study helped ensure that the ultrasound parameters chosen for human trials would be sufficient to reach the brain through the human skull, while promoting a therapeutic environment of cell proliferation and repair, and crucially remaining within established safety standards.

### 3.4. Findings from In Vivo Preclinical Studies

In vivo preclinical studies demonstrate that ultrasound delivered to the brain is effective at clearing AD pathologies, restoring memory functions, and modulating neural oscillations across various animal models. A focus in preclinical research is the use of ultrasound to reduce the burden of toxic protein aggregates. FUS using a 450 kHz single element transducer fixed to the skull with a 100 ms PD, 10% DC, 1 Hz PRF, 1 s PRI, 1.75 I_SPPA_, and 0.175 W/cm^2^ I_SPTA_ for 30 min PTD has been shown to reduce both the number, area, and average size of Aβ plaques in the hippocampal region in 14-month old 5×FAD mice coronal brain sections, compared to age-matched controls. Collected plasma from the lateral saphenous vein and later posterior vena cava confirmed an increase in Aβ_1-42_ levels post FUS, suggesting dissociation and subsequent clearance into the bloodstream [40]. However, a study in the APP23 mouse model suggests that Aβ clearance depends upon the use of intravenously injected microbubbles and the subsequent increased permeability of the blood–brain barrier to achieve clearance [55]. Bobola et al. [51] demonstrated that 40 Hz pulsed ultrasound alone (2 MHz carrier frequency, 40 Hz PRF, 400 µs PD, and an I_SPPA_ of 190 W/cm^2^) can reduce total Aβ plaque burden by 47.4% across the brain and by 28.1% specifically in the CA1 region of the hippocampus after only five days of daily 1 h treatments. This rate of clearance is notably faster than many pharmacological interventions that require months to achieve similar results [60,61,62]. In addition to Aβ, one study demonstrated that low-intensity ultrasound delivered by a 0.5 MHz unfocused piezoelectric transducer with a 50 ms burst at a 5% DC and a PRF of 1 Hz for a sonication time of 15 min at an ultrasound pressure of 0.3 MPa (I_SPPA_ of 6 W/cm^2^) daily for a total of 28 days significantly reduced the expression of both Aβ and Tau protein in the hippocampus and cortex of APP/PS1 transgenic mice [44].

Yang et al. [44] found that 28 days of low-intensity tFUS significantly improved spatial learning and memory, where following ultrasound stimulation, APP/PS1 mice showed shorter latency times to find the platform in the Morris water maze and were performing comparably to wild-type controls by the fourth day of training. Similarly, tFUS treatment brought anxiety behaviour in AD mice back to levels akin to healthy control groups. In fear conditioning tests, tFUS caused a short-term rebound in fear memory after four days of stimulation, though freezing behaviours eventually levelled off to match control groups by the end of the 28-day course [44]. Together, these finding suggest that characteristic aspects of AD pathology—from the protein to the cognitive level—can be improved by ultrasound stimulation.

AD mice are significantly less sensitive to sensory stimuli, such as forepaw electrical stimulation (FES), compared to healthy mice due to impairments in neurovascular coupling and global brain network dysfunction. Li et al. [45] demonstrated that tFUS increases the peak value of cerebral blood oxygenation metabolism in AD mice, bringing their physiological response closer to that of healthy mice. tFUS significantly modulated neurovascular coupling (NVC) in both time and frequency domains by reducing the coupling strength between theta/gamma bands and blood oxygenation, correcting dysregulations caused by AD pathology. This finding indicates that tFUS does not simply increase cerebral blood flow indiscriminately, instead it likely restores the efficiency of the neurovascular unit. By attenuating the aberrant coupling strength between theta/gamma oscillations and hemodynamic responses in AD, tFUS appears to re-establish a more physiological metabolic state.

Our analysis suggests that, across multiple parameter paradigms, ultrasound has the capacity to restore memory function and redress learning deficits in diverse AD mouse and rat models. Treated animals show significantly better performance in the Morris water maze, active place avoidance (APA), and Y-maze tests [40,43,44,57]. In some studies, cognitive improvements remained stable for up to five days after the final stimulation session, suggesting persistent neuroplastic changes [57]. Interestingly, these beneficial effects are apparent across a range of AD models, not just limited to widely used genetic models. In aluminium-induced AD rat models, for instance, low-intensity pulsed ultrasound (LIPUS) prevented cognitive dysfunction and attenuated cerebral damage such as karyopyknosis [31]. This likely reflects a fundamental interaction between basic AD pathological features and ultrasound stimulation and not some artefactual effect of ultrasound due to specific features of a particular pre-clinical model.

Brains affected by AD exhibit significant dysregulation and impairment of specific neural activity frequency bands and synchronisation patterns. These disruptions are considered a hallmark of the disease and are closely linked to cognitive deficits. Recent findings emphasise that ultrasound can directly entrain the brain’s electrical activity. Pulsed ultrasound at 40 Hz (gamma) and 200 Hz (ripple) can restore impaired hippocampal oscillations in AD mice [57]. Gamma-band entrainment has been linked to increased spontaneous gamma power and improved brain connectivity [41]. FUS combined with piezoelectric BCZT nanoparticles injected into the CA3 subregion of the hippocampus of APP/PS1 mice has been used to restore gamma oscillations, essential for higher cognitive functions, and strengthen the hippocampal–medial prefrontal cortex (Hippo-mPFC) network, which is critical for episodic memory. After 7 days of 40 Hz rhythmic stimulation, local field potential recordings showed that gamma power in the AD mice was restored to levels comparable to healthy wild-type controls [50].

In addition to these higher-order effects on neural oscillatory function, ultrasound also induces the production of beneficial biochemical factors linked with neural synchrony and protects cellular longevity. For example, ultrasound treatment upregulates BDNF, GDNF, and VEGF [31,43]. The upregulation of endothelial nitric oxide synthase (eNOS) is identified as a common mechanism that improves cerebral blood flow and promotes remyelination in both AD and vascular dementia models [59]. Interestingly, other aspects of neuroprotection appear to be modulated by ultrasound, where non-invasive ultrasound deep brain stimulation (UDBS) was shown to decelerate telomere shortening in the cortex, peripheral blood, and myocardial tissue of AD and aging mice [56].

A significant and potentially paradigm-shifting trend in recent preclinical studies is the discovery that memory can improve without a reduction in Aβ plaque burden. Repeated treatment at a carrier frequency of 1 MHz improved spatial memory and functional connectivity in APP23 mice even when Aβ levels remained unchanged [54]. This paradigm was more effective than the lower carrier frequency (286 kHz) typically used in clinical trials, suggesting that higher fundamental frequencies may be needed for therapeutic neurostimulatory effects in mice [54]. Furthermore, this finding corroborates that 1 MHz is capable of promoting neurostimulatory effects within the hippocampus itself [21]. Together, these data suggest that ultrasound stimulation can be utilised to reestablish neural function at the network level—effects that could underpin the improvements concurrently observed in cognitive function.

### 3.5. Findings from Clinical Studies

The screened clinical studies focus on the safety, tolerability, and preliminary efficacy of ultrasound as a non-invasive treatment for AD, revealing a consistent focus toward cognitive stabilisation and physiological target engagement via a measurable change in the target tissue’s function or dynamics. Across all clinical studies, FUS treatment was found to be safe and feasible [38,42,48,49,53]. No serious clinical or radiographic adverse events, such as haemorrhages or oedema, were reported across the trials [38,39,42,48,49,53]. High-pressure scanning ultrasound stimulation (SUS) (2.6 MPa), a rasterised iteration of FUS, was found to be painful for some participants, but a titrated dose of 1.95 MPa was well tolerated without severe discomfort [39]. Trials consistently used low carrier frequencies (0.25–1.0 MHz) to ensure ultrasound could penetrate the thick human skull with minimal attenuation [38,39,48].

Clinical outcomes suggest that ultrasound can slow cognitive decline or provide short-term improvements. In randomised controlled trials (RCTs), ADAS-cog scores in treated groups remained relatively stable over 18 months, while scores in placebo groups progressively worsened [38,48]. Multiple studies reported significant increases in MMSE scores and memory performance (immediate recall and recognition) following treatment [49,53]. SUS treatment resulted in statistically significant improvements in behavioural and psychological symptoms of dementia, as measured by the Neuropsychiatric Inventory (NPI), along with a reduction in caregiver distress [39].

Ultrasound induced measurable changes in brain metabolism, blood flow, and structure. PET scans revealed increased regional cerebral metabolic rate of glucose (rCMRglu) in the right hippocampus, indicating that improved memory was positively correlated with these metabolic increases [53]. Furthermore, Arterial spin labelling (ASL) showed a marked increase in relative blood flow (up to 150%) at the targeted hippocampal regions immediately following treatment [42]. Furthermore, automated segmentation showed significant volume increases in the corpus callosum (CC) and lateral orbitofrontal cortex (lOFC). Volume changes in the CC were strongly correlated with improved MMSE scores [58]. Studies have increasingly focused on ultrasound’s ability to remodel brain networks. Repeated low-intensity focused ultrasound (LIFU) to the left dorsolateral prefrontal cortex (DLPFC) significantly enhanced connectivity between the DLPFC and both the perirhinal cortex and dorsomedial prefrontal cortex [49]. SUS treatment induced localised changes in aperiodic EEG content, which suggests a shift in the neuronal balance toward increased inhibition in the treated area [39].

## 4. Discussion

### 4.1. Summary of Evidence

Our scoping search identified 23 relevant studies, spanning a translational spectrum from in vitro cellular models and in vivo transgenic animal studies to human clinical trials. Geographically, this research is emerging rapidly from diverse scientific research hubs including China, South Korea, Australia, and Taiwan, reflecting a global scientific consensus on the potential of this modality.

The synthesised data indicate that FUS is a pleiotropic intervention capable of targeting multiple hallmarks of AD simultaneously, rather than acting on a single pathological target. Unlike previous reviews that have largely focused on the use of FUS in conjunction with microbubbles to permeabilise the blood–brain barrier (BBB) for drug delivery [63], our review specifically isolated studies utilising FUS for direct neuromodulation. The evidence suggests that even without the addition of therapeutic agents or BBB disruption, FUS exerts profound biological effects. In preclinical models, FUS demonstrated consistent efficacy in clearing Aβ and Tau pathology, restoring spatial memory function, and correcting dysregulated neural oscillations. Mechanistically, these effects appear to be mediated through diverse synergistic pathways, including the mechanical disruption of protein aggregates, the immunomodulation of microglial polarisation toward a neuroprotective phenotype, and the upregulation of critical neurotrophic factors. Next steps within the field will likely involve determining the precise cellular and molecular mechanisms responsible for these effects.

Clinically, the data support the safety and feasibility of FUS in patients with AD, with no reports of serious adverse events such as intracranial haemorrhage or vasogenic oedema, which are common concerns in pharmacological anti-amyloid therapies. While efficacy endpoints in humans are currently preliminary, the data suggest significant potential for cognitive stabilisation, arresting the decline observed in placebo groups, and measurable improvements in cerebral metabolism and functional connectivity.

### 4.2. Mechanisms of Action

#### 4.2.1. Mechanical Disruption and Immune Modulation

A significant and novel finding from the in vitro data is the ability of FUS to mechanically disassemble Aβ aggregates through acoustic physics rather than chemical affinity. Unlike monoclonal antibodies that rely on biochemical binding, FUS exerts acoustic radiation forces that can physically alter the conformation of toxic proteins. Studies demonstrated that specific parameters reduce the β-sheet structure of Aβ fibrils by over 55% and dissociate neurotoxic oligomers by approximately 65%. This suggests that FUS acts as a physical catalyst, breaking down dense, insoluble fibrils into smaller, less toxic fragments that are more easily cleared by the brain’s waste disposal systems. Innovative approaches using piezoelectric materials have further expanded this mechanistic repertoire. The use of BiOCl nanosheets, for instance, creates a sonodynamic effect where ultrasound waves induce the generation of ROS. These ROS oxidise specific amino acid residues on Aβ, effectively weakening the hydrogen bonds and hydrophobic interactions that hold the plaque structure together. This represents a cutting-edge convergence of materials science and neurostimulation.

Crucially, FUS appears to bridge the gap between mechanical clearance and biological clearance. The mechanical fragmentation of plaques is followed by a robust immune response. By shifting microglia from the pro-inflammatory M1 phenotype—associated with the release of cytokines like TNF-α—to the phagocytic M2 phenotype, FUS not only reduces neuroinflammation but actively recruits the brain’s innate immune system to phagocytose debris. This is accompanied by an increase in anti-inflammatory markers such as IL-10. This dual mechanism, where mechanical fragmentation renders targets accessible followed by immune activation, may explain the rapid reduction in plaque burden observed in vivo, which often exceeds the clearance rates of pharmacological interventions.

#### 4.2.2. Oscillatory Entrainment and Functional Connectivity

Beyond the plaque clearance paradigm, this review highlights the critical electrophysiological dimension of FUS therapy. AD is increasingly understood as a disease of neuronal disconnection characterised by disrupted neural oscillations, particularly in the gamma band (30–100 Hz), which is essential for binding sensory information and memory consolidation [64]. In vivo studies demonstrated that pulsed ultrasound (particularly at 40 Hz) can directly entrain these rhythms, thereby restoring gamma power and reducing Aβ load. These findings align with the emerging concept of oscillatory therapies in AD. However, unlike sensory entrainment (e.g., flickering lights) which must pass through the visual cortex [65,66,67], FUS offers the advantage of delivering the 40 Hz pulse directly to deep brain structures like the hippocampus. The restoration of the hippocampal–medial prefrontal cortex (Hippo-mPFC) network and improvements in neurovascular coupling further support the hypothesis that FUS restores global brain connectivity. By re-synchronising these neural circuits, FUS may be restoring the cognitive network that has been desynchronised as a result of disease pathology.

#### 4.2.3. The Plaque-Independent Hypothesis and Neuroprotection

Perhaps the most interesting finding identified in this review is the observation that functional and memory improvements can occur independently of Aβ plaque reduction. Leinenga et al. demonstrated that higher-frequency stimulation (1 MHz) improved spatial memory in mice without significantly altering amyloid load. This challenges the Amyloid Cascade Hypothesis as the sole driver of cognitive deficit and suggests that FUS may offer immediate symptomatic benefits to AD patients.

The therapeutic benefit of FUS in these plaque-independent contexts appears to be driven by synaptic plasticity and neuroprotection. The main driver of these therapeutic effects was identified by a consistent upregulation of key growth factors. Furthermore, the upregulation of eNOS helps improve cerebral blood flow, addressing the vascular component of dementia. Remarkable findings regarding the deceleration of telomere shortening in the cortex of aging mice suggest that FUS may even exert anti-aging effects at the cellular level. This implies that symptomatic relief via neuromodulation is achievable through synaptic repair and vascular support, even if disease-modifying clearance of protein aggregates is incomplete.

### 4.3. Clinical Translation and Safety

The transition from rodent models to human application presents unique challenges, primarily due to the attenuating effects of the human skull, which is significantly thicker than that of murine models. Preclinical optimisation using human umbilical vein endothelial cells (HUVECs) helped establish that specific acoustic pressures (0.05–0.5 MPa) are required to upregulate neurotrophic factors without causing cytotoxicity, providing a vital translational bridge across species.

The clinical studies reviewed confirm that current protocols typically using lower frequencies of 0.25–1.0 MHz to penetrate the skull are safe. The recently published International Transcranial Ultrasonic Stimulation Safety and Standards consortium (iTRUSST) guidelines suggest that the risks associated with tFUS are minimal when specific thresholds are adhered to. Mechanical risks are considered nonsignificant provided that the Mechanical Index (MI) and its tissue-corrected counterpart MI_tc_ remain at or below 1.9. Similarly, thermal risks are deemed negligible if the absolute tissue temperature does not exceed 39 °C when assuming a baseline of 37 °C, or if the cumulative thermal dose in the brain is kept below 2 Cumulative Equivalent Minutes at 43 °C (CEM43). Furthermore, thermal safety is maintained if exposure durations adhere to established thermal index guidelines [68].

Importantly, the included studies found that adverse events were minimal, and the procedure was well tolerated, with only minor reports of discomfort at higher pressures that were easily managed by dose titration. Unlike antibody therapies, which carry a risk of Amyloid-Related Imaging Abnormalities (ARIA) [69], FUS without microbubbles appears to spare the neurovascular unit from such damage.

Benefits of tFUS in humans, while early, are promising. The observation of increased regional cerebral metabolic rate of glucose (rCMRglu) in the hippocampus and increased volume in the corpus callosum and orbitofrontal cortex provides objective biomarker evidence that FUS successfully engages target tissues. These structural and metabolic changes correlate with stabilisation in cognitive scores (e.g., MMSE, ADAS-cog), suggesting that tFUS is inducing some form of neuroplasticity rather than merely a placebo effect.

### 4.4. Challenges in tFUS Interpretation

As tFUS gains prominence as a non-invasive neuromodulation technique, rigorous control conditions are essential to distinguish direct neurostimulation from secondary sensory effects. Indeed, tFUS can unintentionally activate peripheral sensory systems, particularly the auditory pathway, which may confound the interpretation of behavioural and electrophysiological data [70,71,72]. To ensure the validity of future findings, experimental designs must account for these artifacts through specific control measures.

In order to mitigate the confound of tFUS eliciting widespread cortical activity resembling that of audible sound, investigators should consider waveform optimisation, whereby the standard rectangular pulse waveform envelope is smoothed, thereby eliminating auditory brainstem responses (ABRs) while preserving direct motor neuromodulation outcomes [61]. Furthermore, experiments should compare neural responses to ultrasound (US) against known auditory stimuli, otherwise known as auditory masking, and other sensory inputs such as air puffs or light flashes to identify overlapping activation patterns. If the spatiotemporal pattern of US activation mirrors that of audible sound, an auditory mechanism is likely involved.

Researchers have successfully utilised genetically deaf mice which can serve as robust controls, confirming that responses persist in the absence of auditory function. While chemically deafening with the use of aminoglycosides has been used to reduce US responses, caution must be taken when considering the potential central neurotoxic effects that, in turn, could confound results [73].

Beyond auditory artifacts, other biological and physical parameters require careful control. The nature of some US-induced movements is highly dependent on anaesthetic depth. In the case of motor outputs, light anaesthesia preserves reflexes that may be mistaken for direct motor cortex stimulation. Therefore, in animal studies, monitoring and reporting of anaesthetic levels is crucial for reproducibility.

Controls involving uncoupled transducers have, in the absence of a coupling medium, demonstrated that airborne sound is not the primary cause of auditory activation and that the mechanism involves fluid or tissue conduction [70,71]. This is especially important when considering the generation of standing waves. Indeed, standing waves can significantly alter the pressure field within the skull, particularly at lower frequencies (<1 MHz) [74]. Because the impact of standing waves varies with head geometry, simulations based on MRI scans of the specific subject are essential to predict the actual pressure field location and standing field ratio.

Moving forward, tFUS studies will likely go beyond simple sham conditions to include active controls that isolate the mechanism of action. By implementing smoothed waveforms, using deafened animal models or auditory masking, and accounting for head geometry and anaesthetic depth, researchers can more reliably attribute observed effects to direct ultrasound neuromodulation.

### 4.5. Strengths and Limitations

This review captured a diverse array of therapeutic modalities, explicitly excluding those focused on BBB opening to isolate the direct neuromodulatory effects of ultrasound. Ultrasound-induced BBB opening remains a promising and effective avenue to deliver therapeutic agents to specific sites in the brain, but a direct focus on ultrasound neuromodulation allows for a clearer understanding of how acoustic energy interacts with neuronal tissue. Given that the BBB in both aged and AD patients is already compromised [8], future studies investigating the delivery of therapeutic agents to AD brain via permeabilisation of the BBB should consider the associated risks of potentially exacerbating AD disease progression. BBB disruption must be carefully controlled so not to induce neurovascular insult and pericyte injury. Importantly, increased permeability at the level of the hippocampus may cause further neurotoxicity by influx of albumin and other systemic proteins which could contribute to a compounding functional deficit in synaptic signalling and metabolism and subsequent structural failure and hippocampal atrophy [8].

There continues to be significant heterogeneity in sonication parameters, e.g., frequency, duty cycle, intensity, duration across the included studies, making direct quantitative comparisons or meta-analyses difficult. It is critical that future studies clearly report all details of protocols and ultrasound parameters used to achieve published effects, thereby reducing risk to participants and providing full transparency. This can be achieved effectively by strict adherence to the ITRUSST consensus on standardised reporting for transcranial ultrasound stimulation [75].

Furthermore, while rodent models allow for detailed histological analysis, approaches to directly translate stimulation paradigms from rodents to humans remain to be fully resolved, where the most fundamental difference of skull geometry poses challenges in selecting the most appropriate acoustic parameters. Additionally, the use of anaesthesia in animal models may confound electrophysiological results, as anaesthetics can dampen neural firing. Finally, larger, longitudinal clinical cohorts are now required to determine if FUS can alter the disease trajectory or merely delay symptoms.

Any articles focusing on transcranial pulse stimulation (TPS) were excluded on the basis that TPS is a similar but distinct modality to that of FUS. However, TPS and FUS are both innovative non-invasive brain stimulation technologies that use sound waves to modulate neural activity. While each modality differs in how each are applied, a comprehensive understanding of the mechanism of action of ultrasound in the context of AD necessitates that TPS be discussed as a promising avenue for non-invasive AD therapeutics despite being excluded from the main body of results.

TPS is a novel, non-invasive neuromodulation technique that utilises navigated, microsecond ultrasound pulses lasting approximately 3 µs [76]. Unlike focused ultrasound techniques, TPS avoids long continuous wave trains, minimising the risk of brain heating and secondary stimulation maxima [76]. It offers high spatial precision, allowing researchers to target specific deep brain networks without the reliance of brain conductivities that may be altered due to pathology [76,77].

TPS significantly improves memory, overall cognition, and depressive symptoms for up to three months and has proven effective for patients ranging from mild to severe AD [77,78,79]. It directly modulates brain wave activity and specifically upregulates functional connectivity in memory and mood networks [76,79,80]. These effects are highly specific to the targeted areas, meaning non-stimulated areas do not receive placebo benefits. Indeed, in addition to functional improvements, TPS is associated with a reduction in cortical atrophy and increased cortical thickness in critical regions like the default mode network [77,81]. TPS represents a highly specific, safe add-on therapy that offers structural, electrical, and functional brain benefits beyond standard symptomatic relief. Refs. [76,81] and therefore, as with FUS, would be highly suitable for further therapeutic exploration.

## 5. Conclusions

A growing evidence base continues to show that FUS is a safe, versatile, and potent neuromodulatory tool for AD. Its therapeutic potential extends far beyond just the mechanical clearance of plaques; it encompasses the restoration of neural oscillations, the modulation of neuroinflammation, the upregulation of neurotrophic factors, and the enhancement of synaptic plasticity. By addressing both the pathological protein aggregates and the electrophysiological network failures characteristic of AD, FUS represents a promising multi-modal intervention. Future research should prioritise the standardisation of acoustic protocols and the integration of advanced neuroimaging biomarkers to fully realise the clinical potential of this non-invasive therapy.

## Figures and Tables

**Table 1:** Characteristics and summary of findings from the included studies.

Author, Year, and Country of Origin	Study Design	Sample Size, Types of Subjects/Participants	Intervention (Control)	Outcome Measures/Indicators	Main Findings
Shimokawa et al., 2022 [38] Japan	Roll-in open trial (safety) and randomised, double-blind, placebo-controlled trial (RCT).	*N* = 5 (roll-in) and *N* = 22 (RCT) patients with early-stage AD (MCI due to AD and mild AD).	Whole-brain LIPUS (0.5 MHz, 1.3 MPa, 32 cycles, 5% duty cycle) for 1 h, 3 times/week. Placebo group received transducer contact without irradiation.	ADAS-J cog scores (primary), CDR sum of boxes, MMSE-J, MRI for safety.	LIPUS was safe with no adverse effects. In the RCT, the LIPUS group showed a non-significant trend toward suppressed cognitive worsening (*p* = 0.257) at 72 weeks; 50% of LIPUS patients were responders (no worsening) vs. 0% in placebo (*p* = 0.053).
Nestor et al., 2025 [39] Australia	Single-centre open-label pilot study.	12 participants with mild-to-moderate AD (MMSE ≥ 10/30).	SUS at 286 kHz; 1.95 MPa; 10 Hz PRF, 10 ms pulse length, 6 s sonication duration.	Safety/tolerability (primary), ACE-III, ADAS-Cog, Neuropsychiatric Inventory (NPI), EEG, functional MRI.	1.95 MPa dose was safe and well tolerated (initial higher dose of 2.6 MPa not tolerated and abandoned). No cognitive changes were noted, but significant improvements in behavioural symptoms (NPI) occurred. EEG showed steeper aperiodic slopes in post-treatment sessions indicating target engagement.
Lee et al., 2026 [40] South Korea	In vitro, ex vivo, and in vivo.	5×FAD transgenic mice (11-month-old and 14-month-old).	FUS using 450–500 kHz carrier frequency transducers (I_SPPA_ 1.22–5 W/cm^2^). In vivo stimulation applied 10 times over 2 weeks (10% DC, 1 Hz PRF, 100 ms pulse duration).	Aβ plaque number/size, ThT fluorescence, PICUP/gel electrophoresis, plasma A beta 42 levels.	FUS alone mechanically dissociated synthetic Aβ fibrils and oligomers. In mice, it significantly reduced plaque number and size in hippocampus/thalamus and increased plasma Aβ by 65.91%, suggesting plaque clearance via perivascular drainage.
Park et al., 2021 [41] South Korea	In vivo	12 male 5×FAD mice (6 treatment, 6 sham) and 6 wild-type controls.	300 kHz carrier frequency TUS 40 Hz PRF, I_SPPA_ 1.2 W/cm^2^; sonication profile consisting of 200 ms ON and 800 ms OFF, repeated (10 s ON and 30 s OFF), 2 h daily for 2 weeks. Sham group received no pulse.	Soluble/insoluble Aβ levels (ELISA), Aβ plaque count, EEG gamma power, cross-frequency phase-amplitude coupling (PAC).	40 Hz TUS reduced total and insoluble Aβ in pre- and infra-limbic cortex and hippocampal plaque counts. Spontaneous EEG gamma power increased and PAC normalised, suggesting functional connectivity improvement.
Nicodemus et al., 2019 [42] USA	Open-label clinical study.	11 patients (*n* = 11 AD) aged 40–95.	Focused transcranial Doppler (2 MHz carrier frequency, 520 mW/cm^2^ targeting hippocampus, once per week for 1 h, for 8 weeks).	Safety/tolerability, cognitive function, motor function tests, MRI for perfusion.	Treatment was safe with no adverse events. 62.5% of patients showed improvement in at least one cognitive measure. MRI imaging showed significantly increased blood perfusion in targeted hippocampal regions post-treatment.
Su et al., 2025 [43] Taiwan	In vivo	96 mice (Male and Female); WT and J20 hAPP transgenic mice.	LIPUS (1 MHz carrier frequency, 1 Hz PRF, I_SPTA_ 528 mW/cm^2^, 5% duty cycle, 3 × 5 min sonication, separated by 5 min rest, daily for 30 days). Control: gaseous anesthesia only.	Morris water maze (MWM) for spatial memory; Thioflavin-S for Aβ plaque burden; immunofluorescence for microglial recruitment (Iba1); Western blot for BDNF, TNF-α, and synaptophysin.	LIPUS significantly improved spatial learning and memory. It reduced hippocampal Aβ plaque burden (0.33% vs. 0.53%) by promoting microglial recruitment to plaques. Observed mechanisms included downregulation of TNF-α and upregulation of BDNF and synaptophysin in CA3 and DG, preserving synaptic integrity.
Yang et al., 2024 [44] China	In vivo	36 female mice (12 WT, 24 APP/PS1 transgenic mice).	TUS (0.5 MHz carrier frequency, unfocussed, 5% duty cycle, 1 Hz PRF, 0.3 MPa (I_SPPA_ 6 W/cm^2^) for 15 min/day over 28 days). Control: AD group without stimulation.	Fear conditioning and MWM for behaviour; EEG for phase-amplitude coupling (PAC); Spike firing rate of interneurons (IN) and pyramidal neurons (PN); ELISA for Aβ and Tau.	Long-term TUS improved cognitive behaviour and reduced Aβ and Tau protein expression. It improved the PAC of delta-epsilon, delta-gamma, and theta-gamma bands. Mechanistically, TUS attenuated the excitability (firing rate) of interneurons and suppressed phase-locked angle deflection in AD mice.
Li et al., 2024 [45] China	In vivo	32 mice (8 WT, 24 APP/PS1 transgenic mice).	TUS (0.5 MHz carrier frequency, 1 kHz PRF, 30% duty cycle, 0.31 MPa). Control: forepaw electrical stimulation only.	Optical intrinsic signal imaging (HbR); local field potential (LFP); neurovascular coupling (Pearson’s correlation coefficient).	TUS modulated cerebral blood oxygenation and neural activity. It increased the peak value of HbR signals and accelerated peak time in AD mice. TUS significantly modulated theta and gamma frequency relative power and reduced neurovascular coupling strength between neural activity and blood oxygenation metabolism, potentially restoring physiological exchange mechanism.
Jang et al., 2020 [46] South Korea	In vitro and ex vivo	Neuro2a (N2a) cells; brain slices from 5×FAD mice (4 months old).	BiOCl nanosheets (3 mg/mL) combined with ultrasound (ultrasonic cleaning bath, 40 kHz, 70 W) (Control: ultrasound only or BiOCl only).	Thioflavin T (ThT) assay; circular dichroism (CD); AFM/DLS for morphology; NBT/TA/ABTS assays for ROS; ThS staining for ex vivo plaque density.	Sono-activated BiOCl nanosheets generate reactive oxidative species (ROS) such as ⋅OH, ⋅O_2_, and H_2_O_2_. This oxidative stress structurally dissociated Aβ aggregates (fibrils and plaques) by destabilising beta-sheets. Ex vivo, plaque density in AD brain slices was reduced from 121.6 to 25.2 cm^2^.
Shindo et al., 2024 [47] Japan	In vitro	Human umbilical vein endothelial cells (HUVECs); Human temporal bone samples (*n* = 20).	LIPUS using assorted transducer probes (carrier frequencies 0.5, 1.0, 1.875 MHz) and duty cycles (1–40%) (Control: no irradiation).	mRNA expression of VEGF, FGF2, and eNOS via real-time PCR; ultrasound transmittance through bone.	Identified optimal human parameters: 0.5 MHz frequency, 5–20% duty cycle, and 0.15 MPa tissue amplitude (1.3 MPa at probe). These parameters significantly upregulated eNOS, VEGF, and FGF2, promoting angiogenesis and neurovascular repair. Lower frequencies (0.5 MHz) showed better transmittance (~58%) through human temporal bone than 1 MHz.
Fuh et al., 2026 [48] Taiwan	Randomised, double-blind, placebo-controlled clinical trial.	9 mild AD patients (6 TUS, 3 Placebo); MMSE 20–26.	TUS (1 MHz carrier frequency, 100 Hz PRF, 2 ms PD, 20% DC). ~15 min per day (3 × 5 min sessions, 5 min rest interval), 5 days per week for six weeks.	ADAS-cog-11 and MMSE scores; NPI-Q; CDR-SB; MRI for safety; TCD for blood flow velocity.	Safety confirmed (no structural brain disorders or BBB disruption). TUS group showed significant MMSE benefit at 24 weeks compared to placebo (2.2 improvement vs. 3.0 decline). ADAS-cog remained stable in TUS group (0.5 change) vs. placebo (5.0 decline) at 52 weeks, specifically in the memory domain.
Jeong et al., 2025 [49] South Korea	Clinical (pilot study).	10 early-stage AD patients (mean age 72.4 years, MMSE < 25, amyloid positive).	LIFU (250 kHz carrier frequency, 500 Hz PRF, 50% DC, 300 ms sonication duration, I_SPPA_ 3 W/cm^2^) for 6 sessions, targeting the left dorsolateral prefrontal cortex (DLPFC) over 2–3 weeks.	Neuropsychological battery (SNSB-II) including memory domain z-scores; resting-state fMRI (seed-to- voxel connectivity with BA 46).	Memory performance significantly improved (*p* = 0.02). Functional connectivity increased between left DLPFC and left perirhinal cortex/dorsomedial prefrontal cortex. Memory improvement positively correlated with connectivity enhancement (Kendall’s tau = 0.56).
Li et al., 2026 [50] China	In vivo	Early-stage AD mouse models (APP/PS1, 2–2.5 months old); *n* = 3–4 per group.	Rhythmic TUS (842 kHz carrier frequency, 40 Hz PRF, 3 ms pulse width) combined with piezoelectric BCZT nanoparticles targeting the CA3 subregion. Controls: WT mice and TUS without nanoparticles.	c-Fos expression; Local Field Potentials (LFPs) for gamma power and theta-gamma PAC; patch-clamp for mEPSCs and AMPAR/NMDAR ratio; Western blot for pNF-kappa B and GluA1; GluN2A; fMRI.	Restored gamma oscillations and enhanced theta-gamma PAC. Rescued synaptic plasticity (mEPSC amplitude and A/N ratio). Activated pNF-kappa B to restore GluR1 expression. Improved Hippo-mPFC functional connectivity. Reduced Aβ oligomer burden.
Lin et al., 2015 [31] Taiwan	In vivo	SD rats; *n* = 6 per group for behavioural tests, *n* = 4 for biochemical analysis.	LIPUS stimulation (1 MHz carrier frequency, 1 Hz PRF, 5% DC, I_SPTA_ 528 mW/cm^2^) administered daily for 49 days (starting 7 days before AlCl3 administration). Control: AlCl3-only group.	Western blot for BDNF, GDNF, and VEGF levels; Morris water maze (AL/RL) and elevated plus maze (TL) for memory; AChE activity; Aβ protein levels; H&E staining (karyopyknosis), BBB permeability, aluminium concentration in the brain.	LIPUS significantly upregulated BDNF, GDNF, and VEGF expressions. Attenuated Al-induced increases in aluminum concentration, AChE activity, and Aβ levels. Significantly improved memory retention (decreased retention latency and transfer latency in Morris water maze) and reduced hippocampal karyopyknosis.
Bobola et al., 2020 [51] USA	In vivo	5×FAD mice (6-month-old male): Acute (*n* = 5 treated/4 sham); Chronic (*n* = 5 treated/ 5 sham).	tFUS (2 MHz carrier frequency, 40 Hz PRF, I_SPPA_ 190 W/cm^2^); acute (1 h) vs. chronic (1 h/day for 5 days).	Microglial activation (plaque colocalisation), Aβ plaque burden, eNOS expression, EEG.	Acute tFUS at 40 Hz increased microglial plaque colocalisation to 36% (vs. 14% sham); chronic treatment for 5 days reduced Aβ plaque burden by 47.4% across brain slices and 28.1% in CA1.
Lu et al., 2024 [52] China	In vivo	33 mice (6-month-old male APP/PS1 and C57BL/6): control (*n* = 11), AD sham-treated (*n* = 11), AD US-treated (*n* = 11).	Ultrasound stimulation (1 MHz carrier frequency, 40 Hz PRF, I_SPTA_ 294 mW/cm^2^, 10% DC) 30 min/day for 5 days (15 min left side, 15 min right side) vs. sham.	Aβ plaque deposition, M1/M2 microglial polarisation, IL-10/TNF-α levels, TMT-based proteomics, Cx IV activity.	US reduced Aβ concentration and plaque intensity; shifted microglia from M1 to M2 phenotype; increased anti-inflammatory IL-1; enhanced mitochondrial cytochrome c oxidase (Cx IV) activity.
Jeong et al., 2022 [53] South Korea	Preliminary pilot clinical study.	8 human patients with probable AD (7 female, 1 male; mean age 78.1).	tFUS (250 kHz carrier frequency, MI 0.30–0.88, 2 Hz PRF, 4% DC, 180 s sonication duration) to right hippocampus with intravenous microbubbles.	BBB opening (DCE-MRI), regional cerebral metabolic rate of glucose (rCMRglu via FDG-PET), cognitive assessments (SVLT, MMSE).	No evidence of BBB opening was found. However, hippocampal glucose metabolism (rCMRglu) significantly increased and memory (immediate recall/recognition) improved; rCMRglu increases correlated with memory improvement.
Leinenga et al., 2024 [54] Australia	In vivo	35 APP23 mice (11 months old): sham (*n* = 12), 1 MHz (*n* = 12), 286 kHz (*n* = 11); wild-type (*n* = 12).	8 weekly SUS-only, comparing effects of 1 MHz vs. 286 kHz carrier frequency transducers (10 Hz PRF) vs. sham.	Spatial memory (Active Place Avoidance), Aβ burden (ELISA/histology), rsfMRI connectivity, SWATH proteomics.	1 MHz SUS-only Improved spatial memory and increased hippocampal/salience network connectivity without reducing Aβ burden; observed proteomic changes related to synaptic vesicle function.
Leinenga et al., 2019 [55] Australia	In vivo	6 APP23 mice (12–14 months old; 5 female, 1 male).	SUS (1 MHz carrier frequency, 10 Hz PRF, 10% DC, 0.7 MPa, 6 s sonication duration per scanned spot). 5× once per week sessions, vs. untreated contralateral hemisphere.	Aβ plaque load (Campbell–Switzer silver stain), plaque diameter, plaque number, H&E staining.	Ultrasound alone (without microbubbles) was not sufficient to clear amyloid plaques or reduce average plaque diameter/number; exogenously supplied microbubbles are required for Aβ clearance.
Zhang et al., 2023 [56] China	In vivo	46 mice: 6 control (C57BL/6), 20 APP/PS1 (male), and 20 aging (female C57BL/6, 18 months old).	tFUS (500 kHz carrier frequency, 500 Hz PRF, 5% DC, 0.34 MPa), 30 min/day for 14 days vs. sham control.	Telomere length (T/S ratio), Morris water maze (MWM), Fear conditioning, c-Fos staining, Western blotting (WRAP53, TERT, BDNF, PSD-95).	tFUS decelerated telomere shortening in the cortex and myocardium of AD mice and improved spatial learning and memory. It also upregulated the neuroactive ligand– receptor interaction pathway and increased BDNF/PSD-95 expression.
Chen et al., 2025 [57] China	In vivo	48 mice: 12 C57BL/6J and 36 APP/PS1 mice (adult male).	TUS (1 MHz carrier frequency): 40 Hz PRF (TUS1) or 200 Hz PRF (TUS2). 0.36 MPa, I_SPPA_ at 4.3 W/cm, 5% DC, 10 s sonication duration, 1 h total sonication time, vs. Sham control.	Morris Water Maze (MWM), Y-maze, LFP/spike recordings (theta, gamma, ripple bands), sharp wave ripples (SPW-Rs).	Both 40 Hz and 200 Hz TUS improved memory performance. 40 Hz TUS increased SPW-R incidence, while 200 Hz TUS increased both incidence and duration. TUS entrained oscillations and improved theta-gamma phase-amplitude coupling.
Huang et al., 2025 [58] Taiwan	Randomised, double-blind, placebo-controlled clinical trial.	9 subjects (6 treatment, 3 placebo) aged 55–90 years with mild AD (MMSE 20–26).	LIPUS (1 MHz, 500 mW/cm^2^ I_SPTA_, 15 min/session (3 × 5 min stimulation), 5 days/week for 6 weeks (sham helmet control).	MRI (FreeSurfer brain volume segmentation), MMSE scores.	Significant volume increases in the CC and LOFC. CC volume changes were strongly correlated with improved MMSE scores (R = 0.807).
Eguchi et al., 2018 [59] Japan	In vivo	C57BL/6 mice (BCAS model for VaD) and 5×FAD mice (AD model), including eNOS- knockout mice	Whole-brain LIPUS (1.875 MHz carrier frequency, 6 kHz PRF, 90 mW/cm^2^ I_SPTA_). In VAD model: 3 × 20 min sonication, 1-day intervals. In AD model: 11 × 20 min sonication duration, concentrated into 3 sessions (1-day inter-session intervals) with 22 days between stimulation sessions, vs. non-stimulated control.	Y-maze, passive avoidance, CBF (laser speckle), RNA-seq, Aβ plaque load, eNOS expression.	LIPUS markedly ameliorated cognitive impairments and reduced Aβ plaques and microgliosis in AD mice. Effects were mediated by eNOS activation, which upregulated neurotrophins and reduced neuroinflammation.

5×FAD = 5 familial Alzheimer’s disease mutations; ABTS = 2,2′-azino-bis(3-ethylbenzothiazoline-6-sulphonic acid) assay; ACE-III = Addenbrooke’s Cognitive Examination version III; Aβ = Amyloid-beta; AD = Alzheimer’s disease; ADAS-cog = Alzheimer’s Disease Assessment Scale-cognitive subscale; ADAS-J = Alzheimer’s Disease Assessment Scale-Cognitive Subscale Japanese version; AFM = Atomic Force Microscopy; AL = Acquisition Latency; ALCl_3_ = Aluminium Chloride; AMPAR = α-amino-3-hydroxy-5-methyl-4-isoxazolepropionic acid receptor; APP/PS1 = Amyloid Precursor Protein/Presenilin-1; BA 46 = Brodmann Area 46; BBB = Blood–Brain Barrier; BCAS = Bilateral Common Carotid Artery Stenosis; BDNF = Brain-derived Neurotrophic Factor; BiOCl = Bismuth Oxychloride; C57BL/6 = Cold Spring Harbor 1957 Black 6; CA1 = Cornu Ammonis 1; CA3 = Cornu Ammonis 3; CBF = Cerebral Blood Flow; CC = Corpus Callosum; CD = Circular dichroism; CDR = Clinical Dementia Rating; CDR-SB = Clinical Dementia Rating-Sum of Boxes; Cx IV = cytochrome c oxidase 4; DC = Duty Cycle; DCE-MRI = Dynamic Contrast-Enhanced Magnetic Resonance Imaging; DG = Dentate Gyrus; DLPFC = Dorsolateral Prefrontal Cortex; DLS = Dynamic Light Scattering; EEG = Electroencephalogram; ELISA = Enzyme-linked immunosorbent assay; eNOS = Endothelial Nitric Oxide Synthase; FGF2 = Fibroblast Growth Factor 2; fMRI = Functional Magnetic Resonance Imaging; FUS = Focused Ultrasound Stimulation; GDNF = Glial cell line-derived neurotrophic factor; GluA1 = Glutamate Ionotropic Receptor AMPA Type Subunit 1; GluN2A = Glutamate Receptor Ionotropic, N-methyl-D-aspartate Subunit 2A; H&E = Hematoxylin & Eosin; H_2_O_2_ = Hydrogen Peroxide; HbR = Deoxy-hemoglobin; HUVECs = Human Umbilical Vein Endothelial Cells; Iba1 = Ionised calcium-binding adapter molecule 1; IL-1 = Interleukin 1; IN = Interneurons; I_SPPA_ = Spatial peak and pulse-average intensity; I_SPTA_ = Spatial peak and temporal average intensity; LFP = Local Field Potential; LIPUS = Low-intensity pulsed ultrasound; LOFC = lateral orbitofrontal cortex; M1 = Macrophage 1; M2 = Macrophage 2; MCl = Mild Cognitive Impairment; mEPSCs = Mini Excitatory Post Synaptic Potentials; MI = Mechanical Index; MMSE = Mini-Mental State Examination; MMSE-J = Mini-Mental State Examination Japanese Version; MRI = Magnetic Resonance Imaging; mRNA = Messenger Ribonucleic Acid; MWM = Morris Water Maze; N2a = Neuro2A; NBT = Nitroblue tetrazolium; NMDAR = N-methyl-D-aspartate Receptor; NPI = Neuropsychiatric Inventory; NPI-Q = Neuropsychiatric Inventory Questionnaire; O_2_ = Oxygen; OH = Hydroxide; PAC = Phase Amplitude Coupling; PICUP = Photo-Induced Cross-linking of Unmodified Proteins; PN = Pyramidal Neurons; pNF-kappa B = Phosphorylated Nuclear factor kappa-light-chain-enhancer of activated B cells; PRF = Pulse Repetition Frequency; PSD-95 = Postsynaptic density protein 95; rCMRglu = Regional Cerebral Metabolic Rate for Glucose; RCT = Randomised Control Trial; RL = Retention Latency; RNA-seq = RNA sequencing; ROS = Reactive oxygen species; SNSB-II = Seoul Neuropsychological Screening Battery-Second Edition; SPW-Rs = Sharp wave ripples; SVLT = Seoul Verbal Learning Test; SWATH = Sequential Windowed Acquisition of All Theoretical Fragment Ion Mass Spectra; TA = Terephtalic acid; TCD = Transcranial Doppler; TERT = Telomerase Reverse Transcriptase; ThS = Thioflavin S; ThT = Thioflavin T; TL = Transfer Latency; TNF-α = Tumour Necrosis Factor alpha; TUS = Transcranial Ultrasound Stimulation; VaD = Vascular Dementia; VEGF = Vascular Endothelial Growth Factor; WRAP53 = WD repeat-containing antisense to TP53.

## Data Availability

The original contributions presented in this study are included in the article/Supplementary Materials. Further inquiries can be directed to the corresponding author.

## Supplementary Materials

The following supporting information can be downloaded at: https://www.mdpi.com/article/10.3390/brainsci16060570/s1, Table S1: Search strategies across the four electronic databases as of 14 January 2026; Table S2: Inclusion and exclusion criteria used to assess eligibility of studies.

## Abbreviations

The following abbreviations are used in this manuscript:

Aβ	Amyloid-beta
ABR	Auditory Brainstem Responses
AChE	Acetylcholinesterase
AD	Alzheimer’s Disease
ADAS-cog	Alzheimer’s Disease Assessment Scale-cognitive subscale
AKT	AKT Serine/Threonine Kinase
APA	Active Place Avoidance
ARIA	Amyloid-related Imaging Abnormalities
ASL	Arterial Spin Labelling
BBB	Blood–brain Barrier
BDNF	Brain-derived Neurotrophic Factor
CAMKII	Ca^2+^-calmodulin-dependent protein kinase II
CC	Corpus Callosum
CD	Circular dichroism
CEM43	Cumulative Equivalent Minutes at 43 °C
CENTRAL	Cochrane Central Register of Controlled Trials
CxIV	Cytochrome C Oxidase 4
DBS	Deep brain stimulation
DC	Duty Cycle
DLPFC	Dorso-lateral Prefrontal Cortex
eNOS	Endothelial Nitric Oxide Synthase
FES	Forepaw Electrical Stimulation
FGF2	Fibroblast Growth Factor 2
FUS	Focused Ultrasound Stimulation
GDNF	Glial-derived neurotrophic factor
Hippo-mPFC	Hippocampal–medial Prefrontal Cortex
HUVECs	Human Umbilical Vein Endothelial Cells
IN	Interneuron
ISPPA	Spatial-peak Pulse-average Intensity
ISPTA	Spatial-Peak Temporal-Average
iTRUSST	International Transcranial Ultrasonic Stimulation Safety and Standards consortium
LFP	Local Field Potential
LIFU	Low-intensity Focused Ultrasound
LIPUS	Low-intensity Ultrasound Stimulation
LOFC	Lateral Orbitofrontal Cortex
LTP	Long-term Potentiation
MCI	Mild Cognitive Impairment
MI	Mechanical Index
MMSE	Mini-Mental State Examination
MRI	Magnetic Resonance Imaging
MWM	Morris Water Maze
N2a	Neuro2a
Na(v)1.5	Voltage-gated sodium channel alpha subunit 5
NIBS	Non-invasive Brain Stimulation
NPI	Neuropsychiatric Inventory
NVC	Neurovascular Coupling
PAC	Phase Amplitude Coupling
PD	Pulse Duration
PN	Pyramidal Neuron
PRF	Pulse Repetition Frequency
PRI	Pulse Repetition Interval
PRISMA	Preferred Reporting Items for Systematic reviews and Meta-Analyses
PTD	Pulse Train Duration
ROS	Reactive Oxygen Species
rCMRglu	Regional Cerebral Metabolic Rate of Glucose
RCT	Randomised Control Trial
SPW-R	Sharp-wave Ripples
SUS	Scanning Ultrasound Stimulation
TEM	Trasmission Electron Microscopy
tDCS	Transcranial Direct Current Stimulation
tFUS	Transcranial Focused Ultrasound
ThT	Thioflavin T
TMS	Transcranial Magnetic Stimulation
TPS	Transcranial Pulse Stimulation
TRP	Transient Receptor Potential
TUS	Transcranial Ultrasound Stimulation
UDBS	Ultrasound deep brain stimulation
US	Ultrasound
WT	Wild-type
VEGF	Vascular Endothelial Growth Factor
