# Supplementary material for: Transcranial Focused Ultrasound Stimulation for Alzheimer’s Disease—A Scoping Review
Source: Brain Sci. 2026 May 28;16(6):570. doi: 10.3390/brainsci16060570 (PMC13296419; doi:10.3390/brainsci16060570)
Supplement: Supplementary file 1 [file brainsci-16-00570-s001.zip › brainsci-4309374-supplementary.pdf]

Table S1. Search strategies across the four electronic databases as of 14 January 2026

No.	Search term	Results
**OVID Medline**		
No.	Search term	Results
**AD concept**		
1	exp Alzheimer Disease/ or Alzheimer disease.tw. or AD.tw.	276149
**FUS neuromodulation concept**		
2	exp Ultrasonic Waves/ or exp Ultrasonic Therapy/ or ultrasound.tw. or focused ultrasound.tw. or LILFU.tw. or low intensity, low frequency ultrasound.tw.	366728
3	(stimulation or neuromodulation).tw.	673067
4	2 AND 3	6935
**Combined concept**		
5	1 AND 4	69
**OVID Embase**		
No.	Search term	Results
**AD concept**		
1	exp Alzheimer Disease/ or Alzheimer disease.tw. or AD.tw.	482329
**FUS neuromodulation concept**		
2	exp focused ultrasound surgery/ or exp focused ultrasound therapy/ or exp low intensity focused ultrasound/ or exp low intensity pulsed ultrasound/ or exp low intensity ultrasound/ or exp MR-guided focused ultrasound/ or exp pulsed ultrasound/ or exp transcranial magnetic resonance guided focused ultrasound/ or exp ultrasound/ or exp or ultrasound.tw. or focused ultrasound.tw. or LILFU.tw. or low intensity, low exp focused ultrasound surgery/ or exp focused ultrasound therapy/ or exp low intensity focused ultrasound/ or exp low intensity pulsed ultrasound/ or exp low intensity ultrasound/ or exp MR-guided focused ultrasound/ or exp pulsed ultrasound/ or exp transcranial magnetic resonance guided focused ultrasound/ or exp ultrasound/ or exp or ultrasound.tw. or focused ultrasound.tw. or LILFU.tw. or low intensity, low frequency ultrasound.tw.	696205
3	exp neuromodulation/ or (stimulation or neuromodulation).tw.	921152
4	2 and 3	14942
**Combined concepts**		
5	1 and 4	239
**Web of Science**		
1	Alzheimer Disease (All Fields) and focused ultrasound (All Fields) and neuromodulation (All Fields)	42

Cochrane Central Register of Controlled Trials		
No.	Search term	Results
**AD concept**		
1	MeSH descriptor: [Alzheimer's Disease] explode all trees	5810
2	(Alzheimer disease):ti,ab,kw	15502
3	#1 OR #2	15502
**FUS neuromodulation concept**		
4	MeSH descriptor: [Ultrasonic Therapy] explode all trees	1468
5	(low intensity focused ultrasound):ti,ab,kw	178
6	(focused ultrasound):ti,ab,kw	1309
7	(LILFU):ti,ab,kw	2
8	#4 or #5 or #6 or #7	2582
9	(neuromodulation):ti,ab,kw	3867
10	(stimulation):ti,ab,kw	72900
11	#9 or #10	73740
12	#8 and #11	269
**Combined concepts**		
13	#3 and #12	6

Table S2. Inclusion and exclusion criteria used to assess eligibility of studies.

Inclusion	Exclusion
Any published primary studies (preclinical or clinical using quantitative, qualitative or mixed methodology), reporting original data	Non-primary studies: narrative reviews, systematic reviews and meta-analysis, editorials, commentaries, opinion papers, letters, education papers, conference abstracts, protocols, reports, theses or book chapters
Studies on FUS in AD	Studies not reporting data on FUS in AD at all<br>Studies reporting data on FUS but not specific to AD
	Studies reporting data of FUS specifically to AD but not for the purposes of neuromodulation
	Studies reporting data of FUS specifically to AD for the purposes of neuromodulation but via delivery of therapeutic agents through the BBB
	Studies reporting data of other neuromodulation modalities distinct from FUS<br>Overlapping data
